# An Ensemble Classifier with Case-Based Reasoning System for Identifying Internet Addiction

**DOI:** 10.3390/ijerph16071233

**Published:** 2019-04-06

**Authors:** Wen-Huai Hsieh, Dong-Her Shih, Po-Yuan Shih, Shih-Bin Lin

**Affiliations:** 1 Department of Information Management, National Chung Cheng University; Director of Chang-Hua Hospital, Chang-Hua County 51341, Taiwan; dire@chhw.mohw.gov.tw; 2 Department of Information Management, National Yunlin University of Science & Technology, Douliu 64002, Taiwan; bestben@gmail.com; 3 Department of Finance, National Yunlin University of Science & Technology, Douliu 64002, Taiwan; D10424003@yuntech.edu.tw

**Keywords:** internet addiction, ensemble classifier, case-based reasoning, machine learning

## Abstract

Internet usage has increased dramatically in recent decades. With this growing usage trend, the negative impacts of Internet usage have also increased significantly. One recurring concern involves users with Internet addiction, whose Internet usage has become excessive and disrupted their lives. In order to detect users with Internet addiction and disabuse their inappropriate behavior early, a secure Web service-based EMBAR (ensemble classifier with case-based reasoning) system is proposed in this study. The EMBAR system monitors users in the background and can be used for Internet usage monitoring in the future. Empirical results demonstrate that our proposed ensemble classifier with case-based reasoning (CBR) in the proposed EMBAR system for identifying users with potential Internet addiction offers better performance than other classifiers.

## 1. Introduction

The rapid expansion of the Internet has been accompanied by criticisms about its impact, both positive and negative, on society and its users. We have been urged to explore its negative impacts, especially those resulting from excessive use of the Internet, the related physical and psychological problems, and harmful consequences toward significant others [1,2,3,4]. One recurring concern involves users with Internet addiction (IA), whose Internet usage has become excessive, out of control, and disrupted their lives [5]. Ignoring coursework, work, and domestic responsibilities, disruption of relationships, social isolation and withdrawal, depression, anxiety, repetitive stress injuries, disturbed sleep patterns and health problems have all been identified as consequences of unrestricted Internet usage [6,7].

Many diagnostic criteria have been developed to identify users with IA and correct their behavior early. However, it is very difficult to confirm the real identity of users since some personal details might not be disclosed, or may even be disguised when answering these diagnostic questionnaires. The Temporary Internet Files (TIFs) in a PC (personal computer) record the Internet access history of users, and reveal genuine and substantial evidence about a user’s browsing behavior. Therefore, analyzing users’ TIFs had been demonstrated to predict excessive or inappropriate usage [8].

Although Mac and computers with other operating systems have similar TIFs, PCs running Microsoft Windows^®^ (Microsoft, Redmond, WA, USA) are still the most popular platform that people use regularly. Therefore, the aim of this study is to demonstrate how the TIFs in PCs can be used for identifying different degree of IA. A secure Web service-based EMBAR (ensemble classifier with case-based reasoning) system was proposed to classify user’s IA patterns and a real-world empirical case was examined. Due to the preliminary success in anomalous behavior detection on PCs [8], we believe that our study can contribute to the understanding of IA behavior and thus help in modifying the usage patterns to foment reasonable Internet usage.

## 2. Related Work

Over the past several years, clinicians have reported numerous cases of IA. Associated psychopathology studies have subsequently been described in the literature. As Griffiths notes, “excessive use of the Internet may not be problematic in most cases, but the limited case study evidence suggests that for some individuals, excessive Internet use is a real addiction and of genuine concern” [9]. Griffiths further considers IA to be a kind of technological addiction. IA is defined as a psychological dependence on the Internet, regardless of the type of activity once logged on [10]. Some others refer to IA Disorder [11], Internet Pathological Use [12], and Internet Dependency [13] to describe this particular Internet-related behavior. These researchers have emphasized that Internet abuse had characteristic tolerance and withdrawal symptoms similar to those of substance abuse. Others believe that IA is an impulsive control disorder, or even an obsessive-compulsive disorder, but the symptoms overlap with those of substance abuse or behavioral addiction, supporting the notion of IA as an addiction—more precisely, an act of addiction.

Since psychopathology researchers started reporting cases of IA, several researchers have tried to develop diagnostic criteria for this issue. Early research focused on criteria, such as the well described set of diagnostic criteria provided by Goldberg [11] and six criteria developed by Griffiths [9]. Young developed an eight-item Internet Addiction Diagnostic Questionnaire (DQ) and proposed an expanded version of the Internet Addiction Test (IAT) [14,15]. Young suggested that addiction level classification scores for IA could be 20–50 = mild, 51–80 = moderate, and 81–100 = severe. Another example includes the checklist of 10 clinical symptoms developed by Scherer [13]. In Morahan-Martin and Schumacker’s study [5], a 13-question “Pathological use scale” questionnaire was developed. Brenner [16] also developed an Internet-Related Addictive Behavior Inventory (IRABI). Other examples include the Chinese Internet Addiction Scale (CIAS) by Chen and Chou [17], revised-IRABI by Chou and Hsiao [18], and Internet Addiction Scale for Taiwan High School Students (IAST) by Lin and Tsai [19]. Recently, other approaches on diagnostic criteria for IA have also been proposed and are listed in Table 1. Among them, the IAT [15] is the first validated and reliable measure of addictive use of the Internet [20]. Therefore, IAT was selected in this study to measure mild, moderate, and severe IA levels. Nevertheless, since it was difficult to confirm the authenticity of the users answering addiction diagnostic questionnaires, an analysis of the TIFs of user for Internet usage can be very helpful. An earlier study involved the analysis of data on a single computer system by using a self-organizing map (SOM) [21]. Feature extraction of Internet behaviors from TIFs in our study is discussed at Section 3.4.

Mak et al. [30] provided a systematic review on the applications of machine learning methods in addiction research. They revealed that a majority of the recent studies had employed supervised learning, while others employed unsupervised learning or reinforcement learning. Among the supervised learning studies, most studies had used ensemble learning methods or multiple algorithm comparisons. The enrolled reinforcement learning studies used the direct method. These results suggested that machine learning methods, particularly supervised learning, are becoming increasingly popular in addiction psychiatry that provide evidence for medical decisions.

In knowledge reasoning, an ensemble classifier includes several general classifiers and combines their addiction level predictions, whereas CBR attempts to solve problems by reusing previous knowledge about similar situations [31]. Ensemble classifiers may improve the accuracy of classifications, and, CBR methods can learn new cases to update their knowledge base. However, the knowledge model of ensemble classifiers is hard to update, whereas CBR methods lag in the complexity of time elapsed for retrieval. Therefore, we tried to take advantage of these two reasoning systems and propose a novel reasoning system that combines an ensemble classifier with CBR. The ensemble classifier is responsible for predicting the addiction level that a user belongs to. CBR will then retrieve the case in predicted classes if there were inconsistencies in general classifiers’ prediction. Our proposed reasoning system is a breed of dynamic diagnostic solution in knowledge reasoning. When the case is classic, the ensemble classifier will cope with the addiction classification. When the chips are down, CBR will then take its place.

## 3. Methodology

**Figure 1:**
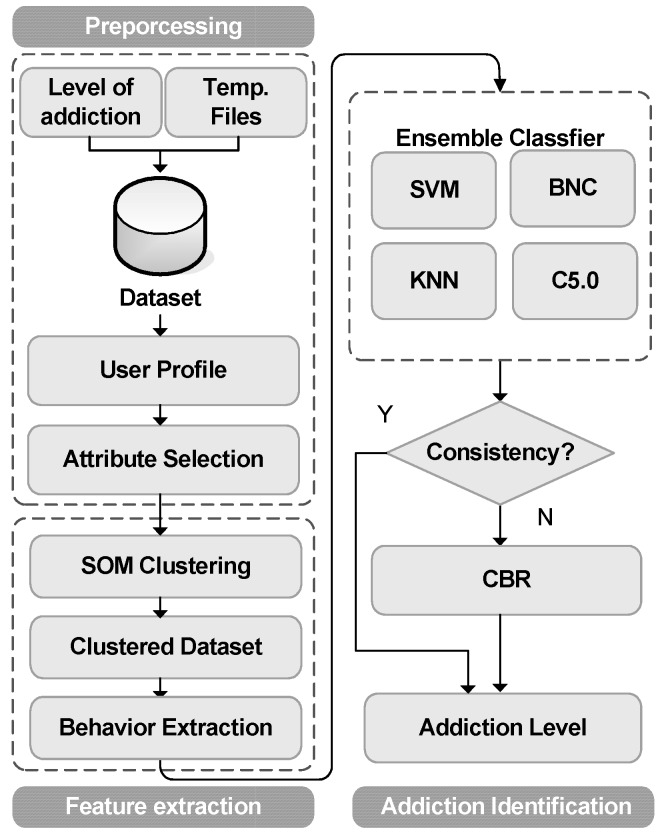
Preprocessing, feature extraction and identification process of Internet Addiction (IA) data.

In order to identify a user’s addiction level, the learning and identification process shown in Figure 1 is proposed. This process is a combination of three phases which are the preprocessing phase, feature extraction phase, and addiction identification phase. In the preprocessing phase, two categories of data are collected from participants. The level of addiction obtained from a user’s IAT questionnaire [15] and users’ TIFs are used for addiction level learning and identification. These data are integrated to create a dataset of user profiles.

The second phase is the feature extraction phase. In this phase, file category, hour and day of the week in TIFs will be transformed into the input vectors of SOM. After clustering of SOM, user’s Internet behavior is extracted. Finally, in the addiction identification phase, an ensemble classifier is used to classify his/her addiction level. The ensemble classifier contains four general purpose classifiers which include support vector machines (SVM) [32], Bayesian network classifier (BNC) [33], k-nearest neighbor (KNN) [34] and decision tree (C5.0) [35]. The learned ensemble classifier model is used to classify a user’s addiction level. If an inconsistency exists, then CBR will step in to make the final judgment. A brief description of SOM, ensemble classifier and CBR follows.

### 3.1. SOM

**Figure 2:**
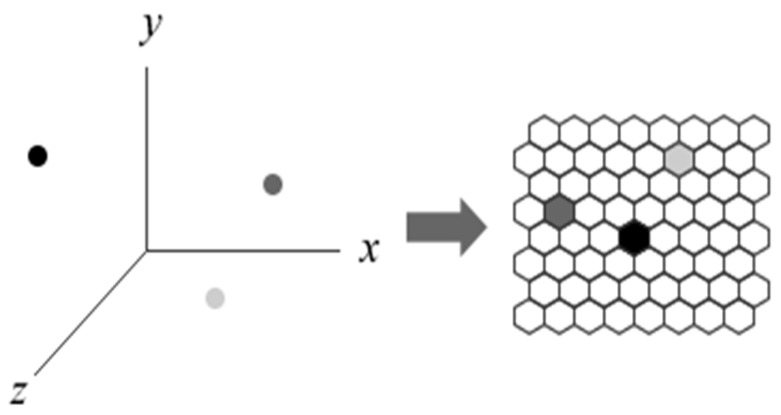
Abstraction of a mapping from three-dimensional data into a two-dimensional self-organizing map (SOM).

Analyzing the TIFs of a single user is cumbersome since the size of TIFs is very large. Therefore, SOM is used to transform high-dimensional TIFs data into a two-dimensional cell space as abstracted in Figure 2. The advantage of using SOM is that all TIF records can be retrieved in a visual two-dimensional space. This feature is particularly important for recognition purposes in our proposed mechanism. The algorithm of SOM [36] is briefly described below.

An *n*-dimensional weight vector *w_k_* is associated with each neuron *k*; *n* is the dimension of the input vector. At each training step, an input vector *x* is randomly selected and the Euclidean distances between *x* and *w_k_* are computed. The input vector on the SOM grid is thus defined as the nearest unit *m_c_* (the best-matching unit, BMU), whose weight vector is closest to the *x*:

(1) $$d=\|x-m_{c}\|=\underset{i}{\min}\left\{\left\|x-m_{i}\right\|\right\}$$

The weight vectors of the BMU and its neighbors on the grid are moved towards the input vector according to the following equation:

(2) $$m_{k}\left(t+1\right)=m_{k}\left(t\right)+h_{ck}\left(t\right)\left[x\left(t\right)-m_{k}\left(t\right)\right]$$

where *h_ck_*(*t*) denotes the neighborhood kernel around the BMU at time *t*. It defines the region of influence that the input sample has on the SOM. The kernel comprises of two parts, the neighborhood function *h*(*d*,*t*) and the learning rate function *α*(*t*):

(3) $$h_{ci}\left(t\right)=\alpha \left(t\right)h_{ci}\left(\|r_{c}-r_{i}\|,t\right)$$

where *r_i_* is the location of unit *i* on the map grid. The learning rate function lies in the interval between 0 and 1.

### 3.2. Ensemble Classifier

**Figure 3:**
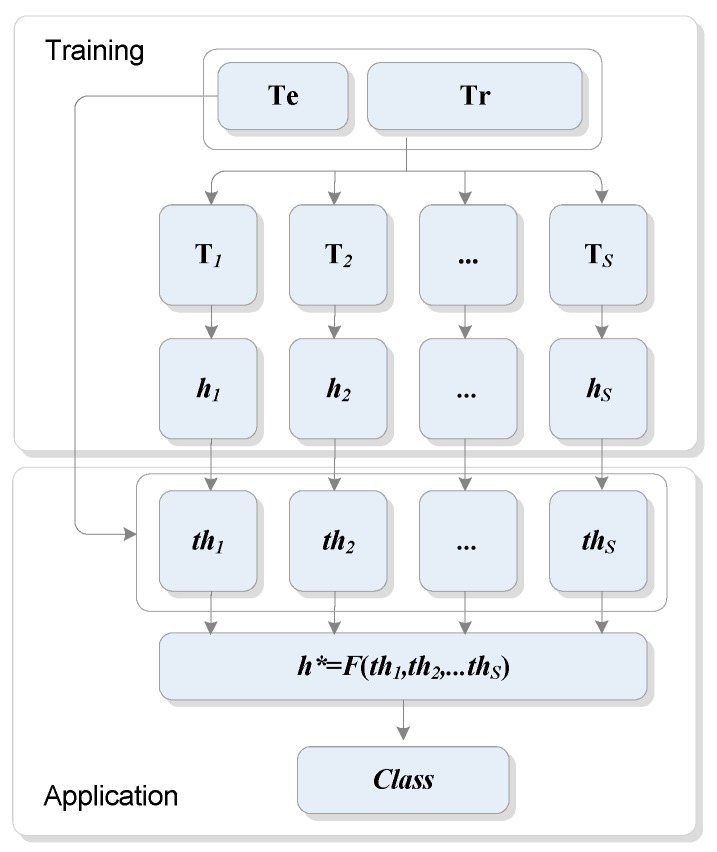
An ensemble classifier with training and application phases.

Figure 3 illustrates the basic framework for an ensemble including *S* general classifiers (*h*_1_, *h*_2_, …, *h_S_*) where *h_j_* is one of the general classifier (*j* = 1, …, *S*). The basic framework includes two parts: (1) Training, and (2) Application. In the Training part, an ensemble E including S general classifiers (*h*_1_, *h*_2_, …, *h_S_*) is generated. One common approach is to form subsets **T***_k_* (*k* = 1, …, *S*) of the initial training set (**Tr**) and then to generate one general classifier *h_k_* for each of them. In the application part, the addiction level predictions of the general classifiers need to be integrated in some way h* = *F* (*th*_1_; *th*_2_; …; *th_S_*) to produce the final classification of the ensemble in the testing set (**Te**) where *th_i_* is the class output belong to *h_i_* classifier (*i* = 1, …, *S*) and h* is a transfer function of the result of all classifier. The most popular techniques used to combine the results of general classifiers are simple voting (also called majority voting) or weighted voting [37].

### 3.3. Case Based Reasoning

CBR is a reasoning paradigm that is able to exploit the information embedded into already solved instances of problems called cases [31,38]. Problem-solving experience is explicitly taken into account by storing past cases in a library, and by suitably retrieving them when a new problem has to be tackled. Case-based problem solving is summarized in the following four steps [38]:

(1) Retrieve the most similar case(s) from the case library;
(2) Reuse them, and more properly apply existing solutions, to solve the new problem;
(3) Revise the proposed solution;
(4) Retain the current case in the library for future problem solving.

With CBR, the system searches for past cases that are analogous to the current case; the solutions of the most similar past cases are then used to create a solution for the current one. The outcome of this redaction technique is a list of cases with its similarity indicator allowing the user to choose from all alternatives [39]. CBR attempts to solve problems by reusing knowledge about previous similar situations [31]. It is an incremental learning process since a new approach is retained each time a problem is solved, making it available for future problems. CBR is useful in searching knowledge, helping users in comparing various tasks and items, automatically notifying users with relevant new knowledge update, and so on [40].

**Figure 4:**
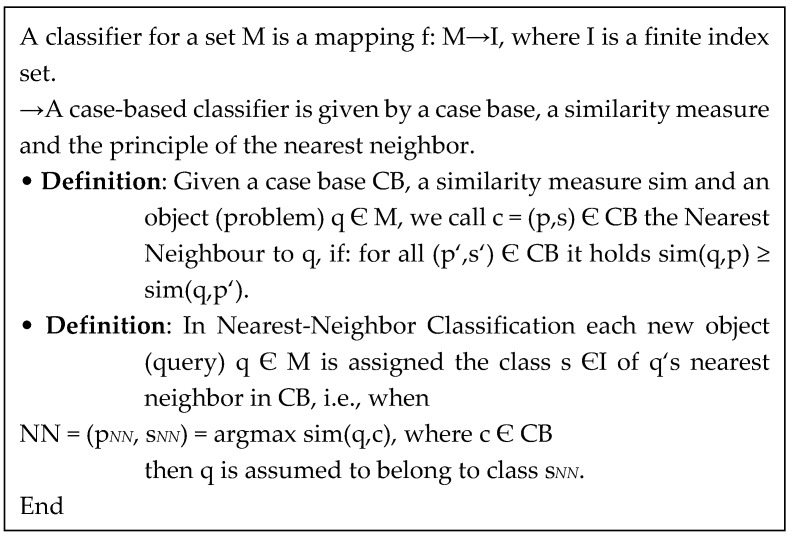
A similarity measure algorithm in CBR.

In this study, a new case is matched against those in the case database to determine the addiction level. A similarity measure is based on the following algorithm listed in Figure 4 and the distribution of variables are all binary.

### 3.4. Addiction Identifying by Ensemble Classifier with CBR

There are two phases in our proposed classification scheme. The first phase is the ensemble classifier classification which has been shown in Figure 3. There are *S* predictions {*th*_1_, *th*_2_, …, *th_S_*} in the first phase. When we input a new test record *x* Є **Te** into the ensemble classifier, the output prediction is determined by the consistency check in the second phase. If there is an inconsistency between general classifiers, the output prediction is determined by the algorithm (in Figure 4) that has the highest similarity with CBR.

**Figure 5:**
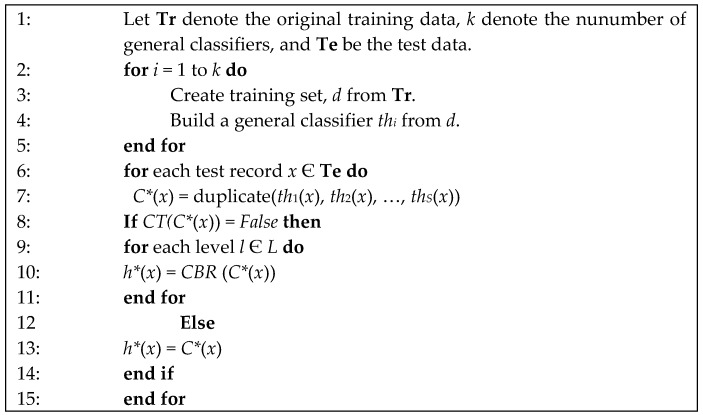
Procedure of ensemble classifier with CBR.

The addiction identification procedure of ensemble classifier with CBR is elaborated in Figure 5, where C* is the duplicate test function of *S* predictions {*th*_1_, *th*_2_, …, *th_S_*} by general classifier and *CT* is a consistency test for ensemble classifier. We’ll then differentiate whether duplicate function test results are the same or not. For example, in the first phase, there is one test record *x* Є **Te** that was predicted by two general classifiers with mild addiction level and moderate addiction level. Since the result is not consistent, it will be analyzed by CBR in the second phase. CBR compares to the records of mild and moderate addiction level, calculated the similarity in these two classes. If similarity of mild addiction level is higher than moderate, then this test record *x* Є **Te** belongs to the mild addiction level.

### 3.5. Feature Extraction of Internet Behaviors

**Figure 6:**
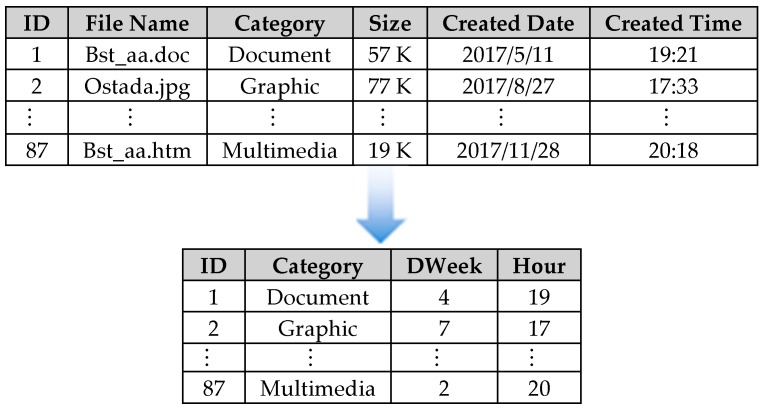
A transform of Temporary Internet files (TIFs) into SOM input vectors.

Temporary Internet Files is a folder on Microsoft Windows which serves as the browser cache to store pages and other multimedia content, such as video and audio files, from websites visited by the user. Fei et al. [8] has demonstrated that analyzing users’ TIFs can be predictive of excessive/inappropriate usage. An example of a user’s TIFs adopted in this study is shown on the top of Figure 6. It includes the users’ logged ID assigned by Windows, downloaded file name, its category and file size, and finally the date and the time it been created (downloaded). All these information is transformed into the input attributes (or vectors) of required SOM as shown at the bottom of Figure 6. It includes the users’ logged ID, file category, day of the week and hour created. The effectiveness of these attributes in representing users’ behavior has been demonstrated by Fei et al. [8].

**Figure 7:**
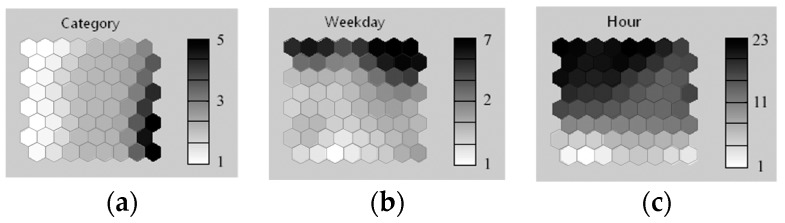
SOM map of one user’s Internet browsing behavior. (**a**) Category; (**b**) Day of the week; (**c**) Hour.

By applying the SOM algorithm, these input vectors is mapped onto a two-dimensional hexagonal grid of size 8 by 8 SOM map as illustrated in Figure 7. SOM map by category (document = 1, graphic = 2, archive = 3, multimedia = 4, others = 5) of Internet access is shown in Figure 7a. Map by day of the week (Monday = 1, Tuesday = 2, …, Sunday = 7) on which the TIFs were created is shown in Figure 7b. Finally, a map by the hour (from 1 to 23) when the TIFs were created (i.e., the hour of day when Internet activities occurred) is shown in Figure 7c. The value of neurons for these three attributes is indicated by gray-level axis on the right side of the corresponding SOM map. The darker neurons represent a higher value in the map. Analyzing the SOM maps in greater detail is useful since it represents the Internet browsing behavior of a specific user.

**Figure 8:**
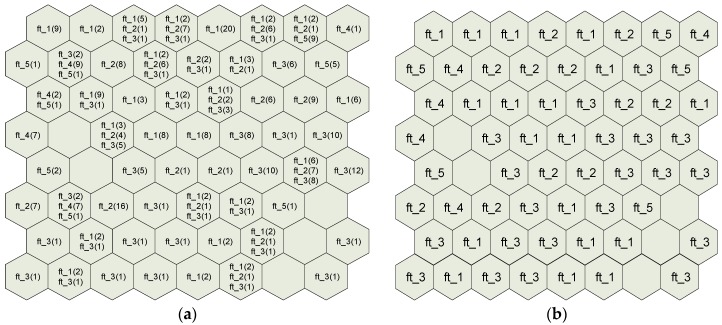
(**a**) File type distribution of Category; (**b**) Extracted Category pattern of one user.

Since the SOM map is different for each individual, we can classify users’ Internet behaviors according to their addiction level. A typical example of the clustered SOM map of Category for one participant is shown in Figure 8a. There are five file types in Category, which are document, graphic, archive, multimedia and others, which are labeled ft_1, ft_2, ft_3, ft_4 and ft_5, respectively. Each cell in the SOM map may contain several clustered duplicate file types with numbered indexes. For example, in the first cell, ft_1(9) indicates that this cell is clustered by the SOM algorithm with file type ft_1 (i.e., document) nine times. Next, each cell is further processed by using Equation (4) and is illustrated in Figure 8b. For example, the third cell of first row in Figure 8a containing three file types (i.e., ft_1(5), ft_2(1) and ft_3(1)) is extracted to ft_1 in Figure 8b since ft_1(5) is the maximum number of file types in this cell:

File_type* = Max{ft_1(*k*), ft_2(*m*), ..., ft_5(*n*)} *Cell(i,j)* = File_type* (4)

where ft_1(*k*), ft_2(*m*), ..., and ft_5(*n*) are in *Cell(i,j)*.

In other words, each cell is represented by its clustered maximum file type. Thus, a SOM feature matrix is created as Figure 8b and this matrix is the extracted feature of a user’s Internet behavior. Then, this SOM matrix is adopted as an input of general classifiers and the output of classifiers are defined by three different addiction levels which is obtained by participant’s questionnaire (IAT) [15]. All the input–output pairs are grouped into a sample set for classifier’s training and testing.

## 4. EMBAR System Overview

**Figure 9:**
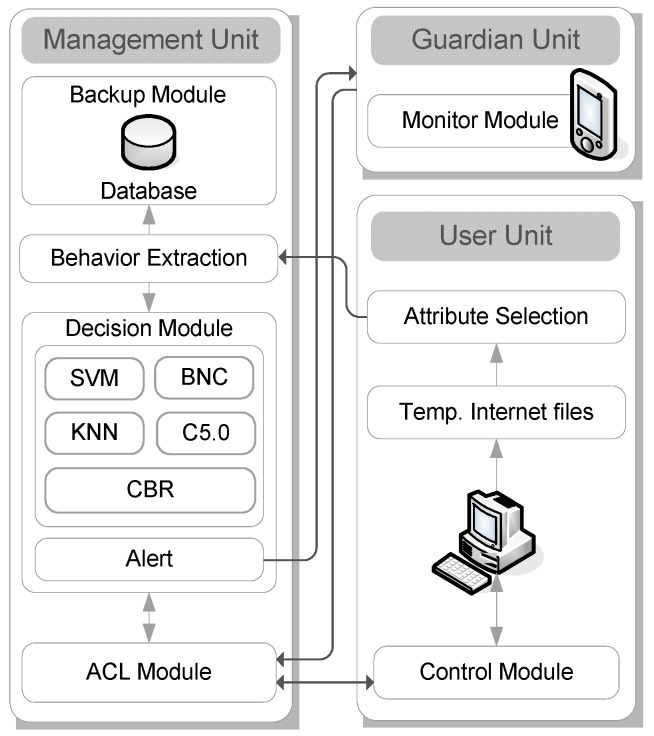
Architecture of the proposed EMBAR system.

In order to diagnose IA, we proposed a secure Web service-based EMBAR system to identify inappropriate Internet usage habits of users. The proposed EMBAR system comprises three units, which are management unit, guardian unit, and user unit. The management unit is the kernel of the EMBAR system; it is responsible for identifying the level of IA, sending an alert message and synchronizing Internet usage with user units. The guardian unit can be a mobile device which traces the status of Internet usage of user anywhere, anytime. The user unit is responsible for extracting the important attributes of TIFs and intervening in Internet usage. The structure of the proposed EMBAR system is shown in Figure 9 and described below.

### 4.1. Management Unit

The Management Unit consists of several modules. Extracted behavior is encoded to the input of decision module. Addiction level of client users is obtained by a decision module. The decision module is responsible for triggering alert messages to the Guardian Unit when the addiction level of a client user is diagnosed to be moderate to severe. When receiving an alert message, the Guardian Unit can then set Internet usage restrictions on the user according to the access control list (ACL) module. Moreover, in order to prevent inadvertent data loss, all records will be backed up regularly by the backup module.

### 4.2. Guardian Unit

The Guardian Unit is responsible for receiving the information from the Management Unit. The information contains addiction alert messages and other statistic data gathered from the User Unit. In order to rectify the inappropriate Internet usage habits, the Guardian Unit can make an intervention schedule through ACL module on Management Unit when a user’s addiction level is reaching moderate or severe levels. The guardian can set ACL from mobile devices even when they’re on the road.

### 4.3. User Unit

The User Unit is responsible for extracting TIF attributes from the user’s computer, and sending them to the decision module in the Management Unit. When the level of addiction is identified, an alert will be sent to a guardian. The User Unit comes with a tightly integrated set of intervention functions, which allows guardians to control users’ access to the Internet. For those who want to restrict users’ Internet access, it allows a guardian to block access to specific websites. For example, a guardian can block a user for certain periods of time; such as after 9 p.m. every weekday. By using it with caution, this EMBAR function can play a very important role in preventing specific user’s addiction tendency. All the access control restrictions will synchronize with the ACL module on the Management Unit.

### 4.4. Web Service Application

**Figure 10:**
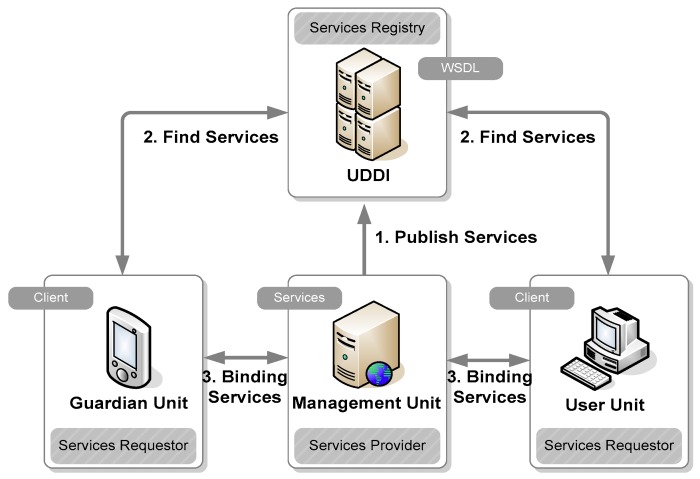
Web service-based EMBAR system.

Web service technology is used to establish the communication infrastructure of our proposed EMBAR system to enhance information integration among distributed, cross-platform, and heterogeneous systems. Our proposed Web service-based EMBAR system is shown in Figure 10.

**Figure 11:**
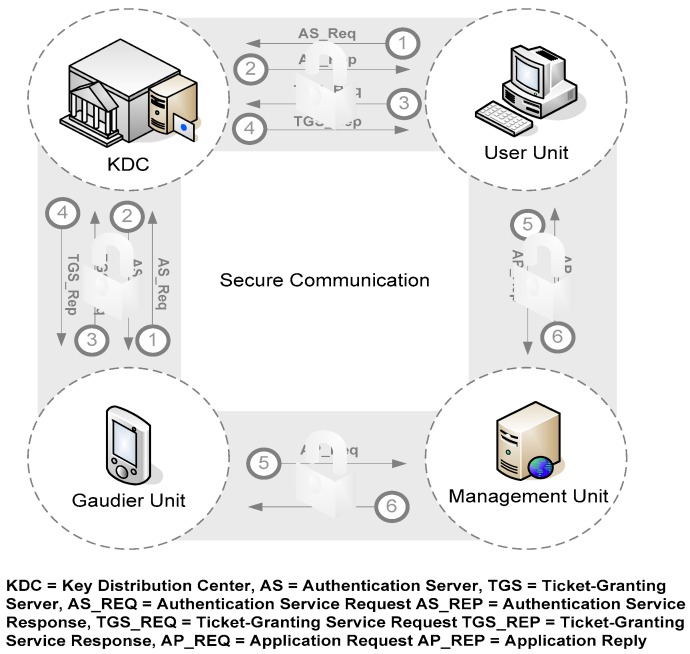
EMBAR system with Kerberos.

**Figure 12:**
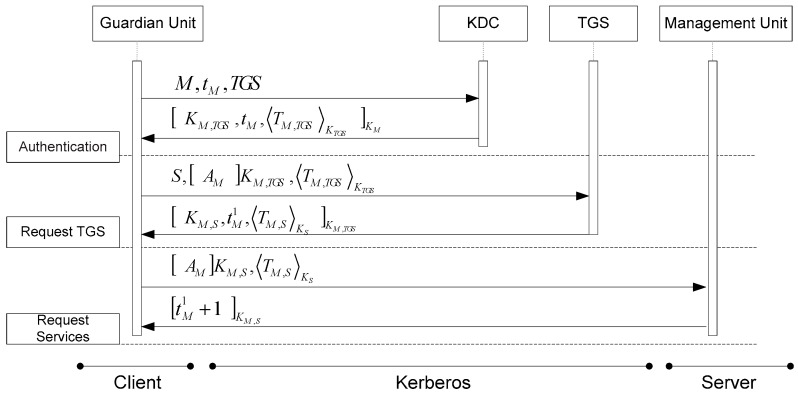
Message flows of an EMBAR system with Kerberos.

In this Web service-based structure, the service provider is the Management Unit and the service requester is the Guardian Unit or User Unit. In order to support a secure environment, our proposed EMBAR system can integrate several security services to satisfy five security requirements as listed in Table 2 [41]. Security services such as Secure Socket Layer (SSL), XML (Extensible Markup Language) Signature [42], XML Encryption and Kerberos [43] are also included for comparison in Table 2. Assume that the entire SOAP (Simple Object Access Protocol) message between client and server has been encrypted and has created a XML signature. An integrated EMBAR system with the Kerberos mechanism is illustrated in Figure 11 and the message flow in abbreviated form is shown in Figure 12 (where M denotes the client and S denotes the server).

In Figure 12 step (1), the Guardian Unit (client M) contacts the Key Distribution Center (KDC) and presents a nonce and requests a ticket for communicating with the Ticket Granted Server (TGS) in KDC. In step (2), the KDC sends the Guardian Unit a session key and communicates with the TGS along with a ticket. The ticket contains a client and TGS session key and is encrypted with the TGS’s secret key. The KDC communicates with the Guardian Unit using a key known only to those two principals. In step (3), the Guardian Unit sends the ticket to TGS along with an authenticator, and requests a key for the Management Unit (server S). The Guardian Unit presents the ticket it obtained from the KDC to the TGS. Then, the TGS generates a session key and a ticket for the Guardian Unit to communicate with the Management Unit. In step (4), the TGS responds to the Guardian Unit with this session key and the ticket, which is encrypted under server’s key. The entire message is encrypted using the Guardian Unit and TGS key. Guardian Unit and Management Unit may go through a process of mutual authentication using their shared key in steps (5) and (6). They then have a session key which can be used in future interactions.

## 5. Results and Discussion

Empirical data were used to examine the performance of our proposal in this section. The test was open to the general public. We had recruited 1328 participants through announcements on national BBS (Bulletin Board System) and national forums from April to October. Among these participants, 603 participants were effective samples according to their questionnaire responses, however, 386 participants refused to provide their TIFs due to privacy concerns. Therefore, a total of 217 remaining participants (47% males and 53% females) were further considered in our analysis. Their average age was 22.08 years with an average 15.54 years of education. All participants completed the questionnaire (IAT) proposed by Young [15], and their TIFs were extracted from their personal computers by a digital forensic tool (Forensic Toolkit, Access Data Corp., Lindon, UK). On average, we spent 2–3 h in collecting one Temporary Internet file per participant, and the time needed for completing this process for all 217 participants was about six months. The proposed classification score for IA by Young [15] is 20–50 = mild, 51–80 = moderate, and 81–100 = severe. Based on this classification there were 114 participants (59 females and 55 males) categorized as mild level, 91 (54 females and 37 males) moderate level participants and 12 participants (three females and nine males) in the severe level according to this classification. The complete collected dataset in a 64 input vector extracted format (SOM size of 8 by 8) is available at http://smartlab.mis.yuntech.edu.tw/.

### 5.1. Procedure

All of our experiments were conducted by randomly dividing the data set into a training set and a testing set, where typically 2/3 of the samples belong to the training set and the remaining 1/3 are the testing set. In addition, since several empirical studies showed that stratified sampling tends to generate comparable results with lower bias and lower variance, a stratified sampling [44] was used in our experiment. The training and testing set are generated in a way that they contain approximately the same proportion of predictor labels as the original dataset. A classification rule is built by applying a classifier to the training set. Then, the samples in the testing set were tested as new samples and were classified by this classification model. This procedure was repeated ten times, and the averages of overall accuracy data were computed.

### 5.2. Performance Criterion

The test performance of the classifiers can be determined by the computation of specificity, sensitivity and accuracy. The experimental IA identification results were evaluated based on the following criteria:

*Specificity*:  (also called the true negative rate) measures the proportion of actual negatives that are correctly identified as such (e.g., the percentage of healthy people who are correctly identified as not having the condition).
*Sensitivity*:  (also called the true positive rate) measures the proportion of actual positives that are correctly identified as such (e.g., the percentage of sick people who are correctly identified as having the condition).
*Accuracy*:  number of correctly classified records/number of total records.

### 5.3. Performance of Different General Classifiers

The classifiers used in our experiment were adopted from an open source software named Waikato Environment for Knowledge Analysis (WEKA). In order to avoid bias, the parameter settings of the four classification models (SVM, BNC, C5.0, and KNN) in WEKA are all by default. Since SVM is a binary classifier, therefore, One-against-the-Rest strategy is used in the experiment. The idea of this strategy is to create an SVM (or another binary classifier) for each category: samples belonging to this category are considered (1), and samples from other categories are considered (−1), so, there is a problem of converting them into binary classifications. In addition, the predetermined clustering number of KNN classifier is 3 due to the IA level is defined to be 3. For SOM, the distance function, decay function and definition of neighborhood used in WEKA plugin are all default too. In order to limit file types in one cell (or neuron), only one file type will stay and the others been filtered in one cell, an 8 × 8 SOM map is adopted in our experiment.

First, we use Category in Figure 6 as an input vector of an 8 × 8 SOM map. After training, the average performance of different general classifiers on the testing set is shown in Table 3. From Table 3, it is clear that severe addiction level is classified with higher accuracy, while others have lower accuracy. The BNC classifier shows the highest performance in mild addiction level, whereas C5.0 shows the highest performance in moderate addiction level. And, SVM has a lower performance in mild and moderate addiction level. Overall, the Bayesian networks classifier and C5.0 demonstrates the highest performance in classification.

Next, we use Created Day (Dweek) in Figure 6 as an input vector of an 8 × 8 SOM map again. The performance of all different general classifiers on testing set in addiction level prediction is not manifested very well. We then used Created Time (Hour) as an input vector instead, and the performance was not good either. After cross examining all participants’ behavior, no matter what addiction level participants belong to, we found that too much timing data concentrating on weekend and after work hours during a day and it is hard to distinguish different addiction levels by using Dweek and Hour as variables. Therefore, in the following section, category is the only choice used as an input vector of an 8 × 8 SOM map.

### 5.4. Performance of Ensemble Classifier with CBR

The weighted voting ensembles experiment is presented in this section. The general classifier weights are determined according to the accuracy of the training set. Each general classifier has its own weight *w_i_* determined by Equation (5):

(5) $$w_{i}=accuracy_{i}/\sum_{i=1}^{k}accuracy_{i}$$

where *k* = the number of base classifier.

**Figure 13:**
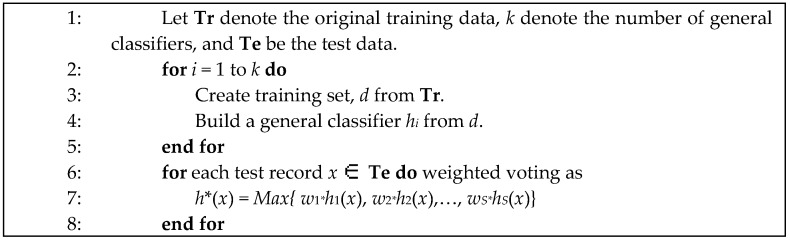
Procedure of the ensemble classifier.

**Figure 14:**
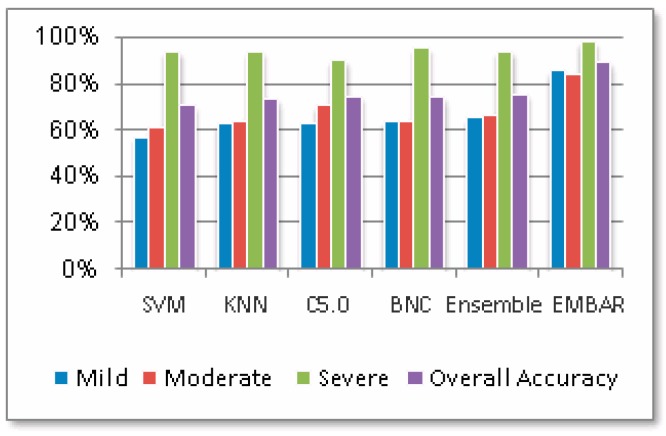
Accuracy percentages of correctly classified instances.

The ensemble classifier procedure is shown in Figure 13. In addition, similarity measurements used in CBR are taken from Figure 5. The experimental results of the ensemble classifier and ensemble classifier with CBR on the testing set are shown in Table 4. In comparison with general classifiers, the weighted voting ensemble classifier shows a better performance as in Table 3. Interestingly, thevoting ensembles classifier has much higher accuracy in mild addiction level and average accuracy. However, the ensemble classifier with CBR has the highest performance. Figure 14 shows the results in graphical form for visual comprehension.

### 5.5. Discussion

The experimental results show that integrating the ensemble classifier with CBR is expected to be the best approach for IA identification. IA level identification accuracies using the ensemble classifier with CBR are 86.3%, 84.9% and 98.6%, respectively. Can it be more accurate? Since CBR has its own limitation of standalone use [45], therefore, integrating an ensemble classifier with CBR would be complementary. Replacing the general classifier with others, such as random forest, may marginally improve the accuracy of a single classifier. We wonder whether there are better alternatives than integrating an ensemble classifier with CBR, and Adaptive Boosting (AdaBoost) may be a feasible alternative [46].

Bagging, Boosting, and AdaBoost are all methods of ensemble learning. The basic condition of ensemble learning is that there should be differences between each classifier, and each classifier must have an accuracy of more than 0.5. If there is no difference in the selected classifiers, it is only classified by many different classifiers, and the results are synthesized without any difference. If the accuracy of the classifier is *p* < 0.5, the classification accuracy decreases as the size of the ensemble increases. If the accuracy is greater than *p* > 0.5, the final classification accuracy rate tends to be 1.

The Bagging concept is to randomly extract (take back and put back, *n* < *N*) samples from the training data to train multiple classifiers (number of classifiers are set by themselves), the weight of each classifier is consistent and the last voting method (majority vote) gets the final result, and this method of sampling is called statistical bootstrap.

The Boosting algorithm synthesizes a number of weak classifiers into a strong one. Unlike Bagging, there is a correlation between classifiers, which go through the error data of the old classifier. The weight is increased, and then the new classifier is trained so that the new classifier learns the characteristics of misclassified data, which in turn improves the classification results. The concept of Boosting is that the old classifier is training some data into confusion. If you use all the raw data to train, the wrong data will stay, so we need to discriminate the wrong information, and the newly trained classifier can get better results for the misinterpreted data. For Boosting, there are two key points. One is how to change the weight of the training data; the other is how to combine multiple weak classifiers into a strong one. There is also a major drawback: the classification algorithm requires prior knowledge of the lower limit of the accuracy of the weak classifier identification.

The AdaBoost algorithm is an improved Boosting classification algorithm. The key is to increase the weight of the classification error samples linearly combined by the first few classifiers, so that each time the new classifier is trained, it will focus on the training samples that are easily classified. Each weak classifier uses a weighted voting mechanism instead of the average voting mechanism. Only weak classifiers with higher accuracy have greater weight. Conversely, weak classifiers with lower accuracy have lower weights. Participants’ IAT scores locate on the boundary of two classes of addiction level in our experiment are frequently seen. Using the AdaBoost algorithm may increase the overall accuracy of identification. However, this study concentrates on the proposal of novel methodology rather than improving accuracy. Therefore, our study is highly informative. After all, there are limitations to our proposed system; it still cannot replace the expert’s intuition and interpretive skills [47,48].

## 6. Conclusions

This study proposed a secure Web service-based EMBAR system for identifying IA using an ensemble classifier with CBR for restraining excessive Internet usage. Analysis of the TIFs discloses evidence about a user’s browsing behaviors, and analyzing them is very helpful to identify possible IA patterns. By using our proposed EMBAR system, guardians can plan activities to rectify the Internet addiction of users under supervision. One promising future research topic may include the analysis of network packets for supervised users. Since TIFs may be modified by a shrewd user, the analysis of data in network packets should be a more appropriate surrogate in identifying IA. Moreover, the analysis of network packets will help us to investigate more advanced Internet-related addiction issues, such as compulsive Internet use and substance use [49], behavioral addictions [50], online auction, online gambling or even in online social media addiction [51].

## Figures and Tables

**Table 1:** Diagnostic criteria.

Researchers	Subject
Khazaal et al. [22]	French Scale (CIUS)
Lee et al. [23]	IAT in Korean
Barke et al. [24]	IAT in German
Widyanto et al. [25]	Psychometric comparison
Tsitsika et al. [26]	Internet gambling
Jelenchick et al. [27]	IAT in US
Brand et al. [28]	Internet sex sites excessive
Guertler et al. [29]	Gamblers

IAT: Internet Addiction Test. CIUS: Compulsive Internet Use Scale.

**Table 2:** Security requirements.

Properties	SSL	XML-S	XML-E	Kerberos	EMBAR
Confidentiality	-	-	Yes	Yes	Yes
Authentication	Yes	-	-	Yes	Yes
Integrity	Yes	Yes	-	Yes	Yes
Non-repudiation	-	Yes	-	-	Yes
Authorization	-	-	-	Yes	Yes

SSL: Secure Sockets Layer; XML: Extensible Markup Language; EMBAR: ensemble classifier with case-based reasoning.

**Table 3:** Accuracy for the general classifiers.

	Mild	Moderate	Severe
(A) SVM			
Sensitivity	72.5%	43.3%	0%
Specificity	39.3%	74.4%	100%
Accuracy	57.5%	61.6%	95.8%
Average Accuracy: 71.6%			
(B) BNC			
Sensitivity	52.5%	76.7%	66.7%
Specificity	78.7%	55.8%	98.5%
Accuracy	64.3%	64.3%	97.2%
Average Accuracy: 75.3%			
(C) C5.0			
Sensitivity	72.5%	56.7%	0%
Specificity	51.6%	81.3%	95.7%
Accuracy	63.0%	71.2%	91.7%
Average Accuracy: 75.3%			
(D) KNN			
Sensitivity	82.5%	40.0%	0%
Specificity	39.3%	81.3%	100%
Accuracy	63.0%	64.3%	95.8%
Average Accuracy (%): 74.4%			

SVM: Support Vector Machine; BNC: Bayesian Network Classifier; C5.0: Algorithm of decision tree, and KNN: k-Nearest-Neighbor.

**Table 4:** Performance of the ensemble classifier and with CBR.

	Mild	Moderate	Severe
(A) Ensemble classifier			
Sensitivity	75.0%	56.7%	0%
Specificity	54.5%	74.4%	100%
Accuracy	65.7%	67.1%	95.8%
Average Accuracy (%): 76.2%			
(B) Ensemble classifier with CBR (EMBAR)			
Sensitivity	87.5%	83.3%	66.7%
Specificity	84.8%	86.0%	100%
Accuracy	86.3%	84.9%	98.6%
Average Accuracy (%): 89.9%			
